# Budding yeast Rad51: a paradigm for how phosphorylation and intrinsic structural disorder regulate homologous recombination and protein homeostasis

**DOI:** 10.1007/s00294-020-01151-2

**Published:** 2021-01-12

**Authors:** Tai-Ting Woo, Chi-Ning Chuang, Ting-Fang Wang

**Affiliations:** Institute of Molecular Biology, Academia Sinica, Taipei, Taiwan

**Keywords:** Rad51 phosphorylation, Mec1^ATR^ and Tel1^ATM^, DNA damage response, S/T-Q cluster domain, Intrinsic structural disorder, Protein homeostasis

## Abstract

The RecA-family recombinase Rad51 is the central player in homologous recombination (HR), the faithful pathway for repairing DNA double-strand breaks (DSBs) during both mitosis and meiosis. The behavior of Rad51 protein in vivo is fine-tuned via posttranslational modifications conducted by multiple protein kinases in response to cell cycle cues and DNA lesions. Unrepaired DSBs and ssDNA also activate Mec1^ATR^ and Tel1^ATM^ family kinases to initiate the DNA damage response (DDR) that safeguards genomic integrity. Defects in HR and DDR trigger genome instability and result in cancer predisposition, infertility, developmental defects, neurological diseases or premature aging. Intriguingly, yeast Mec1^ATR^- and Tel1^ATM^-dependent phosphorylation promotes Rad51 protein stability during DDR, revealing how Mec1^ATR^ can alleviate proteotoxic stress. Moreover, Mec1^ATR^- and Tel1^ATM^-dependent phosphorylation also occurs on DDR-unrelated proteins, suggesting that Mec1^ATR^ and Tel1^ATM^ have a DDR-independent function in protein homeostasis. In this minireview, we first describe how human and budding yeast Rad51 are phosphorylated by multiple protein kinases at different positions to promote homology-directed DNA repair and recombination (HDRR). Then, we discuss recent findings showing that intrinsic structural disorder and Mec1^ATR^/Tel1^ATM^-dependent phosphorylation are coordinated in yeast Rad51 to regulate both HR and protein homeostasis.

## Introduction

During homology-directed DNA repair and recombination (HDRR), DSBs are initially resected to generate single-stranded DNA (ssDNA). This ssDNA is rapidly protected by an ssDNA binding protein complex, RPA, which is subsequently replaced by Rad51 to form a right-handed nucleoprotein filament. This presynaptic filament is essential for homology search and strand invasion. A hallmark of the Rad51 family recombinases from yeast to mammals is that not only are they highly homologous in terms of amino acid sequences, but they also behave similarly in vitro*.* Surprisingly, heterologous expression of fission yeast Rad51 or human Rad51 fails to complement the HDRR defects of the budding yeast *rad51* mutant (Shinohara et al. 1993). Further analyses unveiled that Rad51 recombinases do not act alone in vivo. Rad51 nucleoprotein filaments are regulated via the coordinated actions of diverse Rad51 mediators or interacting partners (Kowalczykowski 2015; Prakash et al. 2009, 2015; San Filippo et al. 2008). It is also noteworthy that Rad51 recombinases and their mediators often undergo a variety of post-translational modifications (e.g., phosphorylation, sumoylation or ubiquitination) in response to DNA damage agents, cell cycle cues or other signaling molecules (Burger et al. 2019; Cremona et al. 2012; Heyer 2015). Protein phosphorylation and dephosphorylation play key roles in many physiological processes and are often deregulated under pathological conditions. This reversible mechanism is mediated by various protein kinases and phosphatases through the addition or removal of a phosphate group (PO_4_^3−^) of polar amino acids, including serine (S), threonine (T), tyrosine (Y) or histidine (H). In the budding yeast *Saccharomyces cerevisiae*, multiple kinases and their transducers are involved in coordinating different HR modules for repairing DNA lesions in mitosis and meiosis (Chuang et al. 2012; Crickard and Greene 2018). Although the strand exchange function of Rad51 is critical for repairing spontaneous DSBs during vegetative growth, it is repressed during meiosis by the meiosis-specific protein Hed1 (Busygina et al. 2008; Tsubouchi and Roeder 2006). Meiotic Rad51 plays a critical role in template choice for HDRR, supporting the strand exchange reaction carried out by the meiosis-specific RecA family protein Dmc1 to repair the programmed DSBs induced by Spo11 (Cloud et al. 2012). Here, we present an overview of Rad51 protein phosphorylation in human and budding yeast. We also discuss recent findings implying that the N-terminal domain (NTD) of yeast Rad51 has dual roles in regulating HDRR and Rad51 homeostasis via its intrinsic structural disorder and through Mec1^ATR^/Tel1^ATM^-dependent phosphorylation.

## Human and yeast Rad51 recombinases are differentially phosphorylated by multiple protein kinases

Human Rad51 has been shown to be phosphorylated by three serine/threonine kinases [checkpoint kinase 1 (Chk1), polo-like kinase 1 (Plk1), casein kinase 2 (Ck2)] and two tyrosine kinases [Abelson tyrosine kinase (c-Abl) and mesenchymal-epithelial transition factor (c-Met)] (Chabot et al. 2019; Narayanaswamy et al. 2016; Popova et al. 2009; Sorensen et al. 2005; Subramanyam et al. 2016; Yata et al. 2012). The phosphorylation sites on human Rad51 and their biological functions in promoting HDRR are summarized in Table 1. The key function of Chk1 and its paralog Chk2 is to relay DNA damage response (DDR) signals from three DNA damage-sensing protein kinases, i.e., ATM (ataxia-telangiectasia mutated), ATR (ATM- and Rad3-Related), and DNA-dependent protein kinase (DNA-PKcs) (Blackford and Jackson 2017; Marechal and Zou 2013). DNA-PKcs are not present in the *S. cerevisiae* genome, whereas Mec1 and Tel1 are the *S. cerevisiae* orthologs of mammalian ATR and ATM, respectively (Craven et al. 2002). c-Abl is phosphorylated and activated by ATM (Wang et al. 2011), whereas c-Met signaling is wired into DDR pathways (Medova et al. 2013). Plk1 plays an important role in the initiation, maintenance, and completion of mitosis (Liu et al. 2017), and it is dephosphorylated and inactivated by protein phosphatase 2A (PP2A) through the ATM/Chk1 DDR pathway (Hyun et al. 2014; Lee et al. 2010). Intriguingly, Plk1 and Ck2 act synergistically during DDR. Plk1 phosphorylates human Rad51 at serine 14 (S^14^), which primes subsequent Ck2-mediated phosphorylation at Rad51 threonine 13 (T^13^) and triggers Rad51 binding to the forkhead-associated (FHA) domain of Nbs1 (Yata et al. 2012). Plk1 also phosphorylates Mre11, a component of the Mre11/Rad50/Nbs1 (MRN) complex, at S^649^ during DDR. Mre11-S^649^ phosphorylation enables subsequent Ck2-mediated phosphorylation at Mre11-S^688^ to impede loading of the MRN complex onto damaged DNA, thereby inhibiting HDRR and premature DNA damage checkpoint termination (Li et al. 2017). Further investigations are needed to reveal the relationship between the Rad51–NBS1 interaction and the formation of Rad51 foci during DDR.

**Table 1:** Summary of protein phosphorylation in human and yeast Rad51

Species	Target site	Kinase	Detection of phosphorylated native (√) or fusion-tagged (Δ) Rad51 proteins in vivo or in vitro (#)	Biological function(s)	References
Human	T^309^	Chk1	Δ (GFP-Rad51)	Flag-Chk1 immunoprecipitates and phosphorylates GFP-Rad51. T^309^ phosphorylation promotes the formation of GFP-Rad51 foci	Sorensen et al. (2005)
Human	S^14^	Plk1	√^a^	S^14^ phosphorylation primes T^13^ phosphorylation	Yata et al. (2012)
Human	T^13^	Ck2	√^a^	T^13^ phosphorylation triggers Rad51 binding to the FHA domain of Nbs1 and the formation of Rad51 foci	Yata et al. (2012)
Human	Y^54^	c-Abl	Δ^a^ (HA-Rad51)	Y^54^ phosphorylation enhances RAD51 nucleoprotein filament formation, and allows RAD51 to compete efficiently with ssDNA binding protein RPA	Popova et al. (2009) and Subramanyam et al. (2016)
Human	Y^315^	c-Abl	Δ^a^ (HA-Rad51)	Y^315^ phosphorylation stimulates Y^54^ phosphorylation	Popova et al. (2009)
Human	T^309^ Y^315^	Chk1 c-Abl	√^a^	Chk1-dependent T^309^ phosphorylation is preferentially stimulated by auto-/paracrine signaling of PLAUR/TLR4 receptor	Narayanaswamy et al. (2016)
Human	Y^159^ Y^191^ Y^205^ Y^315^	c-Met	#^b^ (His_6_-tagged Rad51)	C-Met phosphorylates four tyrosine residues localized mainly in the Rad51 subunit-subunit interface. These modifications might regulate Rad51–BRCA2 interaction	Chabot et al. (2019)
*S. cerevisiae*	GIS^125^EAK VDS^375^PCLP	Cdc28	Δ^d^ (HA-TEV-Rad51) #^e^ (GST-Rad51)	Both S^125^ and S^375^ are required for DNA-binding activity in vitro and homologous recombination in vivo	Lim et al. (2020)
*S. cerevisiae*	S^192^Q	Mec1	Δ^a,c^ (Rad51-Myc, Rad51-TAP)	Ser^192^ is required for Rad51 ATP hydrolysis and DNA-binding activity in vitro and homologous recombination in vivo	Flott et al. (2011)
*S. cerevisiae*	S^2^Q S^12^Q S^30^Q	Mec1 Tel1	√^a^	S^2^, S^12^ and S^30^ phosphorylation act synergistically to enhance Rad51 stability against proteasome-mediated degradation	Woo et al. (2020)

^a^Antisera specific to phosphorylated Rad51 were generated by the corresponding synthetic phosphopeptides and validated by non-phosphorylated Rad51 mutants

^b^The c-Met kinase assays were performed in vitro using recombinant human Rad51 (WT and mutants) and validated by an anti-phosphotyrosine antibody

^c^See Fig. 9 in Woo et al. (2020)

^d^The α-phospho-CDK substrate [Cell Signaling Technology, #2325 (not #23255)] was used to detect phosphorylated HA-TEV-Rad51 protein in vivo. This mAb specifically detects phospho-serine (S*) in PXS*P, PXS*PXR/K or S*PXR/K motifs and it does not react with phospho-threonine- or phospho-tyrosine-containing peptides/proteins

^e^Two recombinant Cdc28 protein kinases (Cdc28-as1-GS and GST-Cdc28) phosphorylate the recombinant GST-Rad51 protein in vitro

Three protein kinases (Mec1^ATR^, Tel1^ATM^ and Cdc28^cdk^) are known to phosphorylate budding yeast Rad51 at different target sites (Table 1) (Flott et al. 2011; Lim et al. 2020; Woo et al. 2020). Cdc28^cdk^, the catalytic subunit of cyclin-dependent protein kinase (CDK), is the master regulator of mitotic and meiotic cell cycles in *S. cerevisiae*. Using a monoclonal antibody specific for phospho-serines (S*) in PXS*P, PXS*PXR/K or S*PXR/K motifs, it was found that Cdc28^cdk^ could phosphorylate S^125^ and S^375^ of an epitope-tagged Rad51 (HA-TEV-Rad51) both in vitro and in vivo (Table 1). Yeast mutant analyses further revealed that mutant non-phosphorylatable Rad51-2A (i.e., Rad51-S^125^A, S^375^A) and Rad51-2E (i.e., Rad51-S^125^E, S^375^E) impair the DNA binding affinity of Rad51 and the Rad51–Rad52 interaction (Lim et al. 2020). Although these results are important, it is noteworthy that the addition of an epitope or fusion protein tag(s) to native Rad51 often results in deleterious impacts to its normal cellular function and/or unexpected post-translational modification(s) (CNC and TFW, unpublished results). Further investigations are necessary to confirm phosphorylation of S^125^, S^375^ and S^192^ (see below) on native Rad51 protein in vivo using antisera targeted specifically against the corresponding phosphorylated peptides. It will also be important to determine if these modifications affect other biochemical or biological properties of native Rad51 in vivo, such as protein stability and nuclear import.

Mec1^ATR^ and Tel1^ATM^ preferentially phosphorylate S/T-Q motifs, i.e., S and T that are followed by glutamine (Q) (Traven and Heierhorst 2005). Mec1^ATR^ alone can perform most of the consolidated functions of Tel1^ATM^ and Mec1^ATR^ in *S. cerevisiae* (Corcoles-Saez et al. 2018; Mallory and Petes 2000; Weinert et al. 1994). Yeast Rad51 contains four S/Q motifs, i.e., S^2^Q, S^12^Q, S^30^Q, and S^192^Q. Mec1^ATR^ is likely responsible for S^192^ phosphorylation, with S^192^ being indispensable for Rad51 adenosine triphosphate (ATP) hydrolysis and DNA-binding activity in vitro as well as HDRR in vivo (Flott et al. 2011). Several lines of evidence indicate that the three S/Q motifs (S^2^Q, S^12^Q, S^30^Q) in yeast Rad51-NTD are authentically phosphorylated in a Mec1^ATR^/Tel1^ATM^-dependent manner (Woo et al. 2020). First, antisera specific to phosphorylated Rad51-S^2^Q, Rad51-S^12^Q and Rad51-S^30^Q peptides detect phosphorylated Rad51 during both vegetative growth and meiosis. Second, no or negligible signals are detected in corresponding antisera of three respective single-amino-acid substitution mutants (i.e., *rad51-S2A*, *rad51-S12A* or *rad51-S30A*), in the phosphorylation-defective mutant *rad51-3A*, and in the *mec1-kd sml1*Δ *tel1*Δ triple mutant. Third, phosphorylation of Rad51-NTD is only moderately diminished in the *tel1*Δ single mutant, indicating that Mec1^ATR^ plays a more prominent role than Tel1^ATM^ in Rad51-NTD phosphorylation. Interestingly, phosphorylation of Rad51-NTD also occurs during vegetative growth in the absence of genotoxin treatments or in sporulating *spo11*Δ diploid cells, but it is not detected in G1-arrested haploid cells or during early meiosis. The reduction of cellular DSB levels during meiosis of the *spo11*-hypomorphic strain (i.e., *spo11-da-HA*) leads to a corresponding reduction in phosphorylation levels of Rad51-NTD without apparent perturbation of steady-state Rad51 protein levels (Woo et al. 2020). Given that Spo11 is the catalytic center where meiotic recombination after the premeiotic S phase is initiated (Keeney 2008), we infer that the robustness of Rad51-NTD phosphorylation is tightly associated with different levels of DNA lesions. It is noteworthy that DSB levels are gradually increased as meiosis progresses in WT yeast (Joshi et al. 2015; Padmore et al. 1991). Accordingly, time-course immunoblots following cycloheximide-shutoff experiments (to establish for how long Rad51 is detectable upon inhibition of protein synthesis) show that Rad51 in early meiotic stages is hypophosphorylated and is indeed less stable than the hyperphosphorylated Rad51 in later meiosis (Woo et al. 2020). Together, these results suggest that spontaneous DSBs are responsible for inducing Mec1^ATR^/Tel1^ATM^-dependent Rad51-NTD phosphorylation during the vegetative S phase and the premeiotic S phase preceding meiotic Spo11-induced DSBs. It is crucial to further decipher if this low-level spontaneous phosphorylation of Rad51 primes for rapid and robust hyperphosphorylation in response to genotoxin treatments or meiotic DSBs in yeast.

## Yeast Rad51 is a paradigm for how the ATR/ATM signaling network regulates both homologous recombination and protein homeostasis

As displayed in Table 1, all known kinases that phosphorylate human Rad51 and yeast Rad51 at various target sites have positive roles in HDRR. Given our recent findings (Woo et al. 2020), summarized below from a mechanistic perspective, Rad51-NTD phosphorylation is unique because its primary role is to enhance Rad51 protein stability by preventing its degradation via the proteasomal pathway. Also noteworthy is that this function can be mimicked by replacing the wild-type (WT) *RAD51* gene with the phosphomimetic mutant (*rad51-3D*) but not with the phosphorylation-defective mutant (*rad51-3A*). Overexpression of WT or even Rad51-3A proteins can also rescue the HDRR defects displayed by the *rad51-3A* and/or *rad51* null (*rad51*Δ) mutants. Cycloheximide-shutoff experiments have further revealed that the half-lives of non-phosphorylated Rad51-3A proteins are ~ 30 min in vivo. In contrast, phosphorylated WT protein and Rad51-3D remain stable for > 180 min (Woo et al. 2020). These differing half-lives of Rad51 proteins readily explain why Rad51 phosphorylation has more profound impacts on Rad51-mediated DNA repair during meiosis than during vegetative growth (Woo et al. 2020), given that the mitotic S phase lasts for 20–30 min (Brewer et al. 1984; Slater et al. 1977) whereas the pre-meiotic S phase during synchronous meiosis of SK1 yeast lasts 65–80 min (Cha et al. 2000; Padmore et al. 1991). In addition, Spo11-induced DSBs take place 1.5–3.5 h after cells have been transferred to the meiosis medium, and the chromosomal foci of recombinases appear and disappear within a single peak (2.5–5 h), with maximum abundance at 3 h (Shinohara et al. 2000), implicating a long period (~ 5 h) when Rad51 is required to repair spontaneous DSBs in pre-meiotic S phase and the subsequent Spo11-induced DSBs (Padmore et al. 1991). The non-phosphorylated Rad51-3A is labile and fails to support DSB repair in *dmc1*Δ *hed1*Δ meiosis (Woo et al. 2020). Thus, higher Rad51 protein stability is required for meiotic DSB repair when Dmc1 is not available. Although the best-known functions of Mec1^ATR^ and Tel1^ATM^ are their roles in mediating DDR, they also have essential functions in regulating protein homeostasis or proteostasis (Corcoles-Saez et al. 2019, 2018). Along with the observations that hyperphosphorylated Rad51 is more stable than hypophosphorylated Rad51 during DDR, we suggest that Rad51 is a paradigm for Mec1^ATR^/Tel1^ATM^-dependent phosphorylation that couples Rad51 homeostasis to HDRR.

## Rad51-NTD displays a nanny function in promoting protein expression

The NTD (1–66 amino acids) of budding yeast Rad51 is unique. Multiple sequence alignments of Rad51 proteins from a variety of model organisms (e.g., fission yeast, *Neurospora crassa*, *Drosophila melanogaster*, *Caenorhabditis elegans*, human, and mouse) have revealed that Rad51-NTD (1–66 amino acids) is specific to the genus *Saccharomyces* (Woo et al. 2020). Yeast mutants (*rad51-*Δ*N*) expressing NTD-truncated mutant proteins still possess the capability of promoting HDRR during both mitosis and meiosis but exhibit much lower efficiency in this function. Consistent with the HDRR-impaired phenotypes, steady-state levels of Rad51-ΔN protein in *rad51-*Δ*N* mutant cells were only ∼ 3% relative to those of WT Rad51. Further analyses demonstrated that Rad51-NTD can act autonomously to promote the expression of an exogenous protein, β-galactosidase (LacZ), with steady-state levels of Rad51-NTD-LacZ being ≥ 13.2-fold higher in vegetative cells than those of LacZ alone (Woo et al. 2020).

Although the highly abundant Rad51 proteins arising from efficient transcription or translation have been correlated with developmental or pathological conditions, such as respectively in mouse embryonic stem cells or irradiation-resistant tumor cells (Raderschall et al. 2002; Tichy et al. 2012), our understanding of how cells secure Rad51 protein stability in various physiological environments is limited (Ahmed et al. 2018; Ning et al. 2017; Woo et al. 2020). It will be critical to verify if other organisms also sustain such highly efficient protein turnover machineries to downregulate Rad51 and/or its nucleoprotein filaments during DDR and if such regulation is also susceptible to counteractions conferred by the yeast Rad51-NTD.

## Intrinsic structural disorder is critical for the nanny function of Rad51-NTD

Many targets of Mec1^ATR^ and Tel1^ATM^ contain at least one S/T-Q cluster domain (SCD), which has been defined as the presence of at least three S/T-Q sites in a stretch of 50 amino acids in *S. cerevisiae* or 100 amino acids in mammals (Cheung et al. 2012; Traven and Heierhorst 2005). Yeast Rad51-NTD contains three SQ motifs that are phosphorylated dependently on Mec1^ATR^ and Tel1^ATM^, so it can be ascribed as an SCD. The best-understood mechanism of SCD phosphorylation involves their association with binding partners harboring a forkhead-associated (FHA) domain (Durocher and Jackson 2002). For example, the human tumor suppressor protein CHK2 has an NH_2_-teminal SCD, followed by an FHA domain and a COOH-terminal catalytic kinase domain. ATR-dependent phosphorylation at CHK2-SCD induces CHK2 activation and phosphorylation-dependent oligomerization via the phospho-SCD/CHK2-FHA interaction (Xu et al. 2002). Moreover, the SCD1 domain (residues 1–29) of the *S. cerevisiae* Rad53 checkpoint kinase contains two adjacent TQ motifs (T^5^Q and T^8^Q) specifically required for recruitment and activation of the Dun1 kinase (Lee et al. 2008). Dun1 phosphorylates Sml1, a potent inhibitor of ribonucleotide reductase (Rnr1), at four serine residues (S^56^, S^58^, S^60^, S^61^), resulting in proteasomal degradation of Sml1 (Andreson et al. 2010). The *sml1* null mutant was originally identified as a suppressor of *mec1* viability (Zhao et al. 1998). Similarly, phosphorylation of the *S. cerevisiae* Hop1 SCD (residues 258–324) at T^318^Q promotes its interaction with the FHA domain of Mek1 protein kinase. Both Hop1 and Mek1 are meiosis-specific proteins essential for HDRR between homologous non-sister chromosomes (Carballo et al. 2008). Notably, it has been shown that there is low sequence complexity in SCDs enriched for S/T-Q motifs (Traven and Heierhorst 2005). Low sequence complexity and high content of S, T, Q, asparagine (N), proline (P), glycine (G) or charged amino acids is a common feature of many intrinsically disordered regions (IDRs) in proteins (Macossay-Castillo et al. 2019; Romero et al. 2001; Uversky 2019). IDRs are known to be involved in folding, proteasomal degradation, molecular recognition, and protein modifications (Tsvetkov et al. 2008; Uversky 2019; Wright and Dyson 1999). Highly-charged IDR protein sequences act as entropic bristles (EBs) that, when translationally fused to partner proteins, enhance water solubility (but not the overall quantity) of the partner proteins (Santner et al. 2012). However, assessments of the steady-state abundance of proteins with IDRs in cells are challenging because they are often proteolytically degraded, yet they sometimes form abnormal aggregates such as disease-causing prions that can persist in cells.

Inspired by these properties of IDRs, we recently reported that, like Rad51-NTD, the IDRs of several other yeast DDR proteins [e.g., Rad53-SCD1, Hop1-SCD and Sml1-NTD (residues 1–50)], as well as non-DDR proteins [e.g., the prion (nucleation) domains of three yeast prion-causing proteins (Sup35, Ure2 and New1) and the NTDs of Vps64 (Far9), Ssk2 and Kel1], possess autonomous and exchangeable activities to enhance high-level protein expression when they are artificially designed as N-terminal fusion tags of LacZ or GFP, among others. We have discovered an interesting correlation between relative LacZ activities and the overall S/T/Q/N percentages in the total amino acid content of these N-terminal IDRs (N-IDRs). Proteome-wide analyses also suggest that such high S/T/Q/N contents in N-IDRs confer a high predicted folding rate on the proteins that carry them. Intriguingly, all the above-mentioned N-IDRs in the non-DDR proteins also possess at least one S/T-Q motif that may be susceptible to phosphorylation by Mec1^ATR^ and Tel1^ATM^. For instance, phosphorylation of Sup35-S^17^Q in response to DNA lesions has been assessed in immunoblots (Chuang et al. 2020). Therefore, we have proposed the “N-terminal intrinsic disorder facilitates abundance” (NIDFA) hypothesis that N-IDRs with high S/T/Q/N contents facilitate protein folding and some could be subject to proteostasis controlled by Mec1^ATR^ and Tel1^ATM^ due to the sporadic emergence of S/T-Q motifs (Chuang et al. 2020). Our NIDFA hypothesis could account for the functions of proteins that harbor an N-IDR but lack binding partners or that fold prior to protein–protein interaction, distinguishing it from two interesting hypothetical models that have been proposed previously. In the first of which, the IDRs in some proteins adopt distinct conformations upon binding to a partner protein, depending on the involvement of different binding partners, chaperones (to support protein folding or degradation) or post-translational modifications (Dyson and Wright 2002; Oldfield et al. 2008; Tompa et al. 2009; Wright and Dyson 1999). These interactions in turn protect IDRs from proteolytic degradation. Alternatively, the “N-terminal folding nucleation” (NFN) hypothesis illustrates that intramolecular interactions modulated by the structured N-terminal domains (SNTDs) that fold spontaneously during protein translation could serve as a nucleation point to organize the as yet unstructured amino acid chain and thus reduce the risk of degradation or aggregation of IDR-containing proteins (Simister et al. 2011). Further investigations must be carried out to delineate if the N-IDRs with high S/T/Q/N content in DDR and/or non-DDR proteins represent docking modules for Mec1^ATR^- and Tel1^ATM^-dependent regulation of protein homeostasis.

## Conclusion and perspectives

Although our knowledge of the post-translational modifications of other DNA recombinase proteins, e.g., RadA, RecA, and Dmc1, is currently limited, findings regarding the post-translational modification of Rad51 have begun to reveal how cells fine-tune the activity of recombinases in HDRR. In addition to regulating the catalytic activity of Rad51, the unique Rad51-NTD in yeast has demonstrated another mechanism by which HDRR can be controlled. In conclusion, *S. cerevisiae* Rad51-NTD possesses dual functions to sustain sufficient levels of Rad51 protein under extreme physiological conditions, such as robust DNA lesions or long periods of DNA repair during vegetative growth and meiosis. As an IDR with high S/T/Q content, Rad51-NTD exhibits autonomous expression-enhancing activity for high-level production of native Rad51 and when fused to exogenous β-galactosidase in vivo. Furthermore, Rad51-NTD is an SCD harboring three putative Mec1/Tel1 target sites. Mec1^ATR^/Tel1^ATM^-dependent phosphorylation antagonizes the proteasomal degradation pathway and further extends the half-life of Rad51. Further investigations are needed to reveal the genetic determinants that control and/or regulate the protein-expression-enhancement function of SCDs in DDR proteins and even other IDRs of non-DDR proteins. Finally, given that > 1000 potential functional IDR segments have been identified in disease-related proteins (Anbo et al. 2019), in conjunction with the implications that deficiencies of ATM and ATR result in Ataxia-Telangiectasia and Seckel syndrome (Shiloh 2001), it is also important to understand whether IDRs or SCDs in human also exert similar functions in coordinating HDRR and protein homeostasis. A rather clear example of the involvement of IDRs in human disease is illustrated by the pathogenic mechanism of Huntington’s disease (HD) (DiFiglia et al. 1997), which is linked to the expansion of a polyglutamine (poly-Q) domain in the N-terminal part of huntingtin protein (Htt) leading to its aberrant aggregation. We propose that the enhanced IDR property conferred by the expanded poly-Q domain may be one of the reasons why Htt aggregates are highly stable, as shown in a yeast HD model (Meriin et al. 2002). Most intriguingly, Mec1^ATR^ has been shown to be essential for relieving the cellular toxicity conferred by Htt aggregation in yeast (Corcoles-Saez et al. 2018), further implying a DDR-independent role of Mec1^ATR^ in modulating the proteostasis of IDR-containing proteins.
