# Correction to: Budding yeast Rad51: a paradigm for how phosphorylation and intrinsic structural disorder regulate homologous recombination and protein homeostasis

**DOI:** 10.1007/s00294-021-01161-8

**Published:** 2021-03-14

**Authors:** Tai-Ting Woo, Chi-Ning Chuang, Ting-Fang Wang

**Affiliations:** Institute of Molecular Biology, Academia Sinica, Taipei, Taiwan

## Correction to: Current Genetic 10.1007/s00294-020-01151-2

The article: Budding yeast Rad51: a paradigm for how phosphorylation and intrinsic structural disorder regulate homologous recombination and protein homeostasis, written by Tai Ting Woo, Chi Ning Chuang, Ting Fang Wang was originally published electronically on the publisher’s internet portal (currently SpringerLink) on 12 January 2021 without open access.

With the author(s)’ decision to opt for Open Choice the copyright of the article changed on 15 February 2021 © The Author(s) 2021 and the article is forthwith distributed under the terms of the Creative Commons Attribution 4.0 International License (http://creativecommons.org/licenses/by/4.0/), which permits use, duplication, adaptation, distribution and reproduction in any medium or format, as long as you give appropriate credit to the original author(s) and the source, provide a link to the Creative Commons license and indicate if changes were made.

The original article was corrected.

